# The role of triggering receptor expressed on myeloid cells-1 (TREM-1) in central nervous system diseases

**DOI:** 10.1186/s13041-022-00969-w

**Published:** 2022-10-22

**Authors:** Chunyan Zhang, Xugang Kan, Baole Zhang, Haibo Ni, Jianfeng Shao

**Affiliations:** 1 Department of Neurology, The Third People’s Hospital of Zhangjiagang City, Suzhou, 215600 Jiangsu China; 2 Department of Neurobiology and Anatomy, XuzhouKeyLaboratoryofNeurobiology, Xuzhou Medical University, Xuzhou, 221004 Jiangsu China; 3 Department of Neurosurgery, The First People’s Hospital of Zhangjiagang City, Suzhou, 215600 Jiangsu China

**Keywords:** TREM-1, Inflammatory response, Central nervous system, Disease

## Abstract

Triggering receptor expressed on myeloid cells-1 (TREM-1) is a member of the immunoglobulin superfamily and is mainly expressed on the surface of myeloid cells such as monocytes, macrophages, and neutrophils. It plays an important role in the triggering and amplification of inflammatory responses, and it is involved in the development of various infectious and non-infectious diseases, autoimmune diseases, and cancers. In recent years, TREM-1 has also been found to participate in the pathological processes of several central nervous system (CNS) diseases. Targeting TREM-1 may be a promising strategy for treating these diseases. This paper aims to characterize TREM-1 in terms of its structure, signaling pathway, expression, regulation, ligands and pathophysiological role in CNS diseases.

## Introduction

Triggering receptors expressed on myeloid cells (TREMs) are a group of receptors expressed on myeloid cells (e.g., monocytes, macrophages, neutrophils). The TREM gene cluster is located on human chromosome 6p21 [1] and has immunoglobulin-like folded transmembrane glycoproteins in the extracellular region that belong to the immunoglobulin superfamily. It is mainly involved in the regulation of inflammation and plays an important role in the innate and adaptive immune response [2]. TREM-1 and triggering receptor expressed on myeloid cells-2 (TREM-2) are two of the most well-characterized members. Both have been studied extensively in CNS disorders, and they are also both expressed on microglia. Although structurally similar, their functions are different. Many studies show that TREM-1 promotes inflammation, while TREM-2 inhibits it. The pathways are partially crosslinked (Table 1). TREM-1 promotes inflammation and inhibits apoptosis through the PI3K/Akt pathway [6], which may lead to a chronic inflammatory state of cells, while TREM-2 inhibits autophagy through this pathway [11]. TREM-1 promotes inflammation through the MEK/ERK and nuclear factor kappa B (NF-κB) signaling pathways [6, 128], while TREM-2 inhibits inflammation by inhibiting these pathways [7, 10]. TREM-1 can enhance the toll-like receptor (TLR)-4-induced inflammatory response, and TLR4 can also increase TREM-1 expression, which may be dependent on the MEK/ERK and NF-κB pathways [28, 94]. Activation of TREM-2 can inhibit the TLR pathway via interleukin-1 receptor activated kinase 3 (IRAK3), and the activated TLR4 pathway can also inhibit TREM-2 expression at the transcriptional level and interfere with TREM-2 by cleaving it into the soluble type [12, 13].

**Table 1:** The similarities and differences between TREM-1 and TREM-2 in CNS diseases

	Similarity	Difference	
		TREM-1	TREM-2
Structure	Composed of three parts: the extracellular immunoglobulin-like domain, the transmembrane domain and the cytoplasmic domain Two subtypes exist: the membrane-bound type and the soluble type [3–5]	Enzyme: MMPs	Enzyme: ADAM 10 and ADAM 17 [5]
Ligands	PAMPs? DAMPs? (still unclear) [6, 7]	HMGB1, HSP70, CD177, actin, PGLYRP1, eCIRP(see “ligands” section)	Cellular debris, lipids, Aβ, nucleic acids, ApoE, LDL [5, 7–9]
Signaling Pathways	The transmembrane domain undergoes signaling by binding to DAP12 containing the ITAM. ITAM is phosphorylated by Src-family kinase, and recruits Syk to trigger downstream signaling cascades, including PI3K/Akt and MEK/ERK [3–5]	Promote inflammation: JAK-STAT3/5, MEK/ERK, PI3K/Akt, NF-κB [6, 128]; Inhibit apoptosis: PI3K/Akt [6]; Promote pyroptosis: NLRP3/caspase-1 [84, 85, 100]; Induce oxidative stress: ROS [84, 127]; Interact with the TLR4 pathway to enhance inflammation [28, 94]	Except DAP12, TREM-2 can also bind to DAP10 [5]; Promote phagocytosis and actin cytoskeleton rearrangement: VAV2/3-Rac1/Cdc42-Arp2/3 [7]; Promote cell growth: Pyk2/β-catenin [7]; Inhibit inflammation: Inhibit NF-κB and MEK/ERK [7, 10]; Inhibit Autophagy: PI3K/Akt-mTOR [11]; Mutual inhibition with the TLR4 pathway [12, 13]

In recent years, growing evidence has shown that TREM-1 plays an important role in the pathology of various neurological disorders, such as ischemic stroke, subarachnoid hemorrhage (SAH), cerebral hemorrhage, glioma, Parkinson’s disease (PD), Alzheimer’s disease (AD), CNS infections, spinal cord injury (SCI) and Spinal cord ischemia–reperfusion injury (SCIRI). In this paper, we provide a general overview of the structure, expression and physiological function of TREM-1 signaling and its role in different neurological disorders.

### TREM-1 structure

Two isoforms of TREM-1 exist: the membrane-bound type (mTREM-1) and the soluble type (sTREM-1). Human mTREM-1 is a type I transmembrane glycoprotein receptor with a relative molecular mass of approximately 30 kDa. It contains 234 amino acids and consists of three parts: the extracellular immunoglobulin structural domain, the transmembrane region, and the cytoplasmic structural domain [3]. The extracellular structural domain is mainly responsible for binding ligands, and the transmembrane region transmits signals intracellularly by binding to the DNAX-activating protein 12 (DAP12) junction protein with a noncovalent bond. sTREM-1 is a glycosylated peptide with only an immunoglobulin-like structural domain, and there are two hypotheses about its origin. The first hypothesis is that the hydrolysis of mTREM-1 by metalloproteinases (MMPs) leads to the shedding of the extracellular region [14]. Weiss et al. [15]. incubated mouse bone marrow-derived neutrophils with unactivated MMP-9 or activated MMP-9. Stimulation of neutrophils with activated MMP-9 decreased mTREM-1 expression and increased sTREM-1 levels compared to unactivated MMP-9. In addition, reduced sTREM-1 expression after inhibition of MMP-9 in a mouse model of coinfection with influenza virus and Streptococcus pneumoniae confirmed that the enzyme cleaves TREM-1. They also proposed that the TREM-1 sequence (MRKAGLWGLLCVFFVSEVKAAIVLEE ERTDLVEQTLTVKCPFNIMKYANSQKAWQR-LPDGKEPLTLVVTQRPFTRPSEVHMGKFTLKHDPSEA-MLQVQMTDLQVTDSGLYRCVIYHPPNDPVVLFHPVRLVVTKGSSDVFTPVIIPITRITERPILITTKYSPSDTTTTRSLPKPTAVVSSPGLGVTIINGTDADSVSTSSVTISVICGIISKSLVFIILFIVTKRTFG) contains a cleavage site of MMP-9 (underlined). The second hypothesis is that it is produced by alternative splicing of the TREM-1 gene [16, 17]. Baruah et al. [17] demonstrated that sTREM-1 is a splice variant of TREM-1. It acts as an endogenous decoy receptor that competes with mTREM-1 for the same ligand and blocks the mTREM-1 signaling pathway.

### The TREM-1 signaling pathway

Activation of the TREM-1 signaling pathway is initiated by the ligands of mTREM-1. Since the receptor has no immunoreceptor tyrosine-based activation motif (ITAM), it needs to be associated with the ITAM-containing adapter protein DAP12 for signaling. Positively charged lysine residues in the transmembrane structural domain of TREM-1 are noncovalently bonded to negatively charged aspartate residues in the transmembrane region of the DAP12 junction protein. Upon binding to DAP12, tyrosine phosphorylation in ITAM mediated by Src-family kinases recruits the zeta-associated protein of 70 kDa (ZAP70) and spleen tyrosine kinase (SYK) [18], which initiates a cascade of downstream signaling reactions, including the phosphoinositide phospholipase C-gamma (PLCγ), phosphoinositide 3-kinase (PI3K), Janus kinase (JAK), and mitogen-activated protein kinase (MAPK) pathways. These pathways induce Ca^2+^ mobilization, actin cytoskeleton rearrangement, and the activation of transcription factors, which are responsible for encoding the expression of cell-surface molecules, proinflammatory cytokines and chemokines [19–22].

In addition to the direct effects induced by TREM-1 signaling, there is growing evidence for crosstalk between TREM-1-induced innate immune pathways and pattern recognition receptors (PRRs), particularly TLRs and nucleotide-binding and oligomerization domain (NOD)-like receptors (NLRs). Activation of TLRs can increase the expression of TREM-1 [23–26]. TREM-1 amplifies the inflammatory response by regulating signaling downstream of PRRs, thus increasing inflammatory factor release and amplifying innate and adaptive immune responses. Myeloid differentiation factor 88 (MyD88), CD14, IL-10, and IL-1β are significantly reduced when TREM-1 expression is decreased in macrophages, whereas the expression of linker proteins and effector proteins in the TLR4 pathway is not significantly affected. The expression of TLR4 is not altered after knockdown of TREM-1, but the TLR4-induced NF-κB pathway is downregulated [27]. Wang et al. [28]. found that mice with high tidal volumes develop ventilation-induced lung injury (VILI), elevated expression of TREM-1, TLR4, MyD88, and NF-κB, and increased levels of the proinflammatory cytokines TLR4, MyD88, and NF-κB after upregulation of TREM-1. The opposite results were observed with the TREM-1 antagonist, suggesting that the exacerbation of TREM-1-induced VILI in mice is associated with the TLR4-MyD88-NF-κB-dependent signaling pathway. In both in vitro and in vivo studies, infection with Aspergillus fumigatus (A. fumigatus) has been shown to upregulate TREM-1 expression on corneal epithelial cells. The TREM-1 antagonist peptide inhibits the elevation of inflammatory cytokines caused by fungal infection, whereas blockade of TLR4 enhances the effect of the TREM-1 antagonist peptide. TLR4 and MyD88 inhibition partially suppress A. fumigatus-induced TREM-1 upregulation, confirming TREM-1 and TLR4 crossover in corneal fungal infections [29]. TREM-1 is not only involved in amplifying TLR signaling but also has a synergistic effect on the recognition of peptidoglycan (PGN)-induced inflammatory responses by NLRs. After moderate stimulation of TREM-1 by NLR ligand, activated TREM-1 can amplify NLR signaling, induce NOD2 expression, and increase IL-1β and IL-6 expression [30].

### Regulation of TREM-1 expression

In addition to myeloid cells, TREM-1 expression has also been detected in nonimmune cells, such as airway epithelial cells [31], corneal epithelial cells [29], gastric epithelial cells [32], hepatic endothelial cells [33], Kupffer cells [34], and hepatic stellate cells [35]. Bosco et al. [36] analysed gene expression profiles and found that in hypoxia, TREM-1 transcript levels are significantly upregulated in monocyte-derived mature dendritic cells (mDCs), thereby inducing the increased secretion of proinflammatory cytokines and chemokines. TREM-1 expression on monocyte-derived immature dendritic cells (iDCs) is also elevated in chronic hypoxia and can be antagonized by cellular reoxygenation [37]. Hypoxia-inducible transcription factors (HIFs) are important transcriptional effectors that regulate the cellular response to hypoxia, and HIF-1a expression is downregulated in iDCs and mDCs during hypoxia [38], presumably because HIF-1a is involved in regulating TREM-1 expression under hypoxia. In addition, 1,25(OH)2D3 regulates the innate immune response of human monocytes and macrophages through the upregulation of TREM-1 expression [39]. Nguyen et al. [40] observed elevated TREM-1 expression in porcine keratin-forming cells after addition of vitamin D. In another study, rapamycin, a specific inhibitor of the mammalian target of the rapamycin (mTOR) signaling pathway, was found to inhibit TREM-1 expression induced by 1,25(OH)2D3, suggesting that 1,25(OH)2D3 induces TREM-1 expression through the mTOR signaling pathway [41]. Prostaglandin E2 (PGE2)-pretreated peripheral blood mononuclear cells were activated, which resulted in increased TREM-1 expression as well as interleukin 8 (IL-8) and tumour necrosis factor alpha (TNF-α) production [42]. Other substances, such as lipopolysaccharide (LPS; TLR4 ligand) [23, 24], lipophosphatidic acid (TLR2 ligand) [25], tryptophanyl-tRNA synthetase 1 (WARS1, TLR2 and TLR4 ligand) [26], and sodium urate [43], can increase TREM-1 expression. However, Molad et al. [44] found that administration of CpG oligodeoxynucleotides (CpG-ODNs) alone did not alter mTREM-1 expression, but the combination of CpG-ODNs with LPS significantly inhibited LPS-induced TREM-1 upregulation, suggesting that the CpG-ODN-induced TLR9 pathway negatively regulates TREM-1 expression on macrophages. In addition, prostaglandin D2 (PGD2), prostaglandin J2 (PGJ2), and 15-deoxy-delta-prostaglandin J2 (15-dPGJ2) can also suppress TREM-1 expression through the activation of nuclear factor erythroid 2-related factor 2 (Nrf2) and the inhibition of NF-κB [45]. Antimicrobial peptides such as LL-37 [46] and LP17 [47] and anti-inflammatory cytokines such as interleukin 10 (IL-10) [48] and transforming growth factor β (TGF-β) [49] are also known to inhibit mTREM-1 activity.

The expression of TREM-1 is regulated by transcription factors (e.g., activator protein 1 (AP-1), NF-κB, PU.1, Nrf2). Recognition of pathogen-associated molecular patterns (PAMPs) and danger-associated molecular patterns (DAMPs) by the innate immune system activates macrophages and microglia, triggering an inflammatory response, and activated macrophages and microglia regulate TREM-1 expression at the transcriptional level. In turn, activation of TREM-1 can also promote macrophage and microglia activation. Binding sites for NF-κB, PU.1 and AP-1 are present in the promoter of TREM-1 [50]. LPS or Pseudomonas aeruginosa (P. aeruginosa) can activate macrophages, and subsequently NF-κB and PU.1 induce the expression of TREM-1 in RAW264.7 cells, and inhibition of NF-κB or overexpression of PU.1 can inhibit TREM-1 expression. Silencing PU.1 increases TREM-1 expression [50]. In addition, Nrf2 also inhibits LPS- and P. aeruginosa-induced TREM-1 expression in RAW264.7 cells [45]. Activation of isolated mouse and human microglia with LPS significantly induces TREM-1 expression, and the same response was observed in acute encephalitis and cerebral ischemia. Inhibition of NF-κB prevents LPS-induced TREM-1 expression [51]. Kupffer cells (KCs) are liver-resident macrophages. Stimulation by HIV and HCV activates KCs and upregulates the expression of proinflammatory cytokines, chemokines, and TREM-1. Activation of TREM-1 by a targeted agonist increases HIV/HCV-mediated macrophage inflammatory responses. Silencing TREM-1 suppresses the inflammatory response induced by HIV/HCV-stimulated macrophages [52]. These studies support that microglia and macrophage activation can increase TREM-1 expression. In vivo and in vitro experiments have demonstrated that sTREM-1 can cause microglial overactivation through the PI3K/AKT signaling pathway, leading to hippocampal damage [53]. In an AD model, TREM-1 facilitated microglial phagocytosis of amyloid beta (Aβ) [54]. Numerous preclinical experiments have demonstrated that TREM-1 inhibition can inhibit macrophage and microglia activation, thereby attenuating the inflammatory response [55]. The above studies suggest that microglial activation interacts with TREM-1 expression and that this action is at least partially dependent on NF-κB signaling.

## TREM-1 Ligands

TREM-1 ligands are elusive, and the molecular mechanisms of receptor‒ligand interactions are poorly studied. In CNS diseases, no clear TREM-1 ligand has been reported to date. Existing studies have suggested that high-mobility group box 1 (HMGB1), PGN recognition protein 1 (PGLYRP1), heat shock protein 70 (HSP70), CD177, actin, and extracellular cold-inducible RNA-binding protein (eCIRP) may be natural ligands for TREM-1, and all of these endogenous ligands are associated with inflammatory responses. HMGB1 is a ubiquitous nuclear protein that acts as a DAMP and activates multiple PRRs that regulate innate and adaptive immune responses. Using immunoprecipitation and surface plasmon resonance (SPR), Wu et al. [34]. observed that HMGB1 can directly interact with TREM-1, but the signal transduction pathway that follows their binding is unclear. HMGB1 can be released by neurons and glial cells and participates in the pathophysiology of many CNS diseases, including blood‒brain barrier disruption and neuroinflammation [56]. It has been reported that damaged neurons after SAH release HMBG1 and HSP70 [57, 58]. The expression of TREM-1 and HMGB1 is increased after ICH, and the two proteins interact to promote inflammatory responses. HMGB1 inhibitor reduces neuroinflammation after ICH. One study suggested that damaged neurons after ICH release HMBG1 and induce inflammatory responses. HMBG1 may be a potential endogenous ligand for TREM-1 [59]. The investigators proposed that there may be a neurotoxic cycle between microglia and neurons. TREM-1-induced microglial pyroptosis leads to the release of inflammatory mediators which damage neurons. The damaged neurons release large amounts of HMGB1 and HSP70, which further activate microglial pyrolysis via TREM-1. However, this hypothesis needs to be verified by further studies. In neurodegenerative diseases such as AD, frontotemporal dementia, Parkinson's disease and multiple sclerosis, HMGB1 released by damaged tissues is considered a risk factor for promoting cellular senescence, memory impairment, chronic neurodegeneration and the progression of neuroinflammation [60, 61]. Under chronic unpredictable mild stress conditions, HMGB1 released from microglia and hippocampal neurons can lead to neuroinflammation, which may cause depression-like behaviour [62]. Since the downstream receptors of HMGB1 also include receptors for advanced glycation end-products (RAGE) and TLRs, it remains to be seen whether the proinflammatory effects of HMGB1 in the CNS are produced through activation of TREM-1.

eCIRP is an endogenous inflammatory mediator that has also been identified as a DAMP that promotes inflammation, tissue damage, and death in systemic inflammation [63–65]. Denning et al. [66]. demonstrated a strong affinity between eCIRP and TREM-1 by SPR, and their immunofluorescence experiments demonstrated the colocalization of eCIRP and TREM-1 on macrophages after recombinant murine CIRP (rmCIRP) stimulation. Furthermore, LP17 reduced rmCIRP-induced systemic inflammation and lung injury and inhibited rmCIRP-induced TNF-α production in RAW264.7 cells, indicating that eCIRP is a novel TREM-1 ligand. These authors also synthesized a novel peptide inhibitor, M3, that could block the interaction between eCIRP and TREM-1 and inhibit the eCIRP-mediated inflammatory response. Borjas et al. [67]. used M3 to treat hepatic ischemia/reperfusion (I/R) mice and found that M3 attenuated hepatic I/R injury by inhibiting TREM-1. Furthermore, the survival rate of the M3-treated group was higher in mice undergoing nonischemic hepatic lobectomy after liver I/R, supporting the importance of TREM-1 and eCRIP in inflammation. In brain-specific inflammation, microglia can express CIRP, but studies on whether eCRIP released from microglia can activate TREM-1 are lacking. Aβ-mediated neural stress is associated with AD. Exposure to Aβ stress increases the release of eCIRP from BV2 microglia. eCIRP mediates neuroinflammation through activation of the IL-6Rα/STAT3/cyclin-dependent kinase-5 (Cdk5) signaling pathway in neurons [68]. In a middle cerebral artery occlusion (MCAO) model, CIRP and TNF-α levels were observed to be elevated. CIRP-deficient mice show significantly reduced brain infarct volume and TNF-α expression and microglial activation. Therefore, CIRP may play a key role in activating microglia and inducing neuroinflammation. In addition, hypoxia has been found to induce CIRP expression, translocation and release from BV2 microglia. eCIRP induces TNF-α expression through the NF-κB pathway, leading to neuronal damage [69]. Alcohol exposure induces microglia to express CIRP and release eCIRP, which acts as a mediator of neuroinflammation causing neuronal damage and death [70]. eCIRP can also mediate alcohol-induced hypometabolism and cognitive impairment in brain regions [71]. Whether eCIRP causes inflammation through activation of TREM-1 in CNS disorders is an important question for future research.

Sharapova et al. [72, 73]. demonstrated that both recombinant innate immune protein Tag7 (also known as PGLYRP1) and HSP70 are potential ligands for TREM-1, and their binding to TREM-1 induces the activation of cytotoxic lymphocyte subpopulations, the latter of which are lethal to major histocompatibility complex-negative tumour cells. HSP70 is also present in necrotic bone marrow cell lysates and is responsible for the production of proinflammatory cytokines [74]. However, direct binding of HSP70 to TREM-1 was not observed in the experiments by Wu et al. [34], possibly because HMGB1 has a stronger affinity than HSP70. However, studies of HSP70 in CNS diseases have shown that it reduces neurodegeneration, stimulates neurogenesis, restores memory, and protects neurons from various types of stress injury. Recombinant HSP70 or HSP70 pharmacological inducers are promising potential drugs for the treatment of ischemic injury and neurodegenerative diseases [75–77]. Given that the protective role of HSP70 in CNS diseases is opposite to the proinflammatory role of TREM-1, it seems unlikely that HSP70 is a ligand for TREM-1 in the CNS. PGLYRP1 is a neutrophil granule protein with antimicrobial properties that forms a TREM-1 ligand complex by binding to PGN. Read et al. [78]. used SPR and flow cytometry to demonstrate that PGLYRP1 and TREM-1 can bind to each other. While PGN can enhance the binding of both, it cannot bind directly to TREM-1. This research suggests that peptidoglycan in the bacterial cytoderm stimulates neutrophil degranulation and that the released PGLYRP1 binds to PGN and induces TREM-1 expression, thereby enhancing the immune response. Colonna et al. [79]. first suggested that CD177 may be an endogenous ligand for sTREM-1. They incubated HEK293 cells transfected with CD177 with sTREM-1 and found that the two were bound to each other, thus supporting this hypothesis. In 2007, Haselmayer et al. [80]. used recombinant soluble fusion protein to demonstrate the presence of a ligand for TREM-1 on human platelets. In 2017, Fu et al. [81]. found that recombinant actin could bind directly to the extracellular structural domain of TREM-1 and enhance inflammation, which could be inhibited by LP17, but the response was not observed in TREM-1-/-mice. Furthermore, they found that actin colocalized with TREM-1 on lung tissue sections from septic mice, suggesting that actin is the TREM-1 ligand present on platelets.

Whether known or other unknown ligands exist in CNS diseases and how they activate the TREM-1 signaling pathway require more research.

### TREM-1 signaling in central nervous system diseases

In recent years, many studies have found that TREM-1 expressed on microglia is involved in the pathological processes of CNS diseases, including ischemic stroke, SAH, cerebral hemorrhage, glioma, PD, AD, CNS infections, SCI and SCIRI (Tables 2, 3). TREM-1 may be a potential target for the treatment of these diseases. Microglia are the major immune cells in the CNS and play an important role in mediating the inflammatory response in CNS diseases. Microglia can recognize and eliminate foreign invaders, repair local tissue damage caused by injury, and play an important role in neural circuit regulation and general homeostasis. Tissue injury activates microglia and induces TREM-1 expression, and elevated TREM-1 further promotes microglial activation and polarization into the proinflammatory phenotype. However, persistent or chronic activation of microglia may lead to irreversible central nervous injury. In addition, innate immune cells generate an immune response in response to pathogen invasion or tissue damage, and the induced inflammatory response is associated with the activation of PRRs. As previously discussed, there is crosstalk between PRRs and the TREM-1 signaling pathway.

**Table 2:** The role of the TREM-1 signaling pathway in CNS diseases

Disease	Study Models	Molecules in the TREM-1 Signaling Pathway	Conclusions	Refs.
Ischemic Stroke	Rat MCAO model/in vitro	ROS, NLRP3	TREM-1 induced microglia activation, oxidative stress and pyroptosis	[84]
	Mouse MCAO model/in vitro	SYK	TREM-1 induced inflammation and pyroptosis	[85]
Subarachnoid hemorrhage	Mouse	SYK	TREM-1 promoted microglia activation and neutrophil infiltration, induced neuroinflammation	[95]
	Rat	TLR4	TREM-1 interacted with the TLR4 pathway to promote EBI	[94]
	Rat	p38MAPK/MMP-9, ZO-1	TREM-1 was involved in blood‒brain barrier disruption and brain oedema	[99]
	Mouse	NLRP3	TREM-1 induced microglia pyroptosis and neutrophil infiltration, promoted neuroinflammation, and led to neuronal apoptosis and degeneration	[100]
Intracerebral hemorrhage	Mouse	PKCδ/CARD 9	TREM-1 regulated microglia polarization and induced neuroinflammation	[59]
Parkinson’s disease	Zebrafish	MAPK	TREM-1 inhibition improved locomotor impairment, suppressed inflammation	[110]
	Rat	MAPK	TREM-1 inhibition inhibited microglia and astrocyte hyperactivation	[110]
	In vitro	MAPK	TREM-1 inhibition suppressed inflammation and enhanced autophagy	[110]
Alzheimer’s disease	Mouse	Aβ	TREM-1 promoted microglia phagocytosis of Aβ, alleviated AD neuropathology and improved AD-related spatial cognitive deficits	[54]
Spinal cord injury	Mouse/in vitro	HO-1	TREM-1 promoted microglia activation, induced inflammation and oxidative stress	[127]
Spinal cord ischemia‒reperfusion injury	Mouse/in vitro	SYK	TREM-1 mediated neuronal apoptosis	[128]

**Table 3:** TREM-1 expression in CNS diseases

Disease	TREM-1 Isoform	Sample	Results	Refs.
Ischemic Stroke	TREM-1	Serum	TREM-1 levels were elevated and correlated with stroke severity	[83]
Post-stroke depression (PSD)	sTREM-1	Plasma	Reduced sTREM-1 levels in patients with PSD were negatively associated with the severity of depressive symptoms	[89]
Subarachnoid hemorrhage	sTREM-1	CSF	Early elevation of sTREM-1 was positively correlated with white blood cells, CRP, and the severity of the disease	[92]
	sTREM-1	Plasma	sTREM-1 levels were elevated and positively correlated with Hunt-Hess classification and CRP levels	[95]
	sTREM-1	CSF	sTREM-1 levels were positively correlated with the Hunt-Hess classification	[93, 94]
Intracerebral hemorrhage	sTREM-1	Serum	Elevated sTREM-1 levels may correlate with the severity of disease	[104]
Alzheimer’s disease	TREM-1	Blood	TREM-1 mRNA levels were elevated	[111]
	sTREM-1	Plasma	Elevated sTREM-1 levels were positively correlated with AD progression and total tau protein levels	[112]
Gliomas	TREM-1	Blood	Patients with TREM-1/TREM-2 ratios greater than 125 had shorter survival times than those with ratios less than 125	[122]
	sTREM-1	Serum	sTREM-1 levels were lower than that of normal subjects	[122]
Bacterial meningitis	sTREM-1	CSF	sTREM-1 levels were elevated	[123]
	sTREM-1	CSF	sTREM-1 levels were elevated and correlated with mortality	[124]
	sTREM-1	CSF	sTREM-1 levels were higher in surviving patients than in deceased patients	[125]
External ventricular drain-related ventriculitis	sTREM-1	CSF	sTREM-1 concentrations were elevated	[126]

### Ischemic stroke

**Fig. 1:**
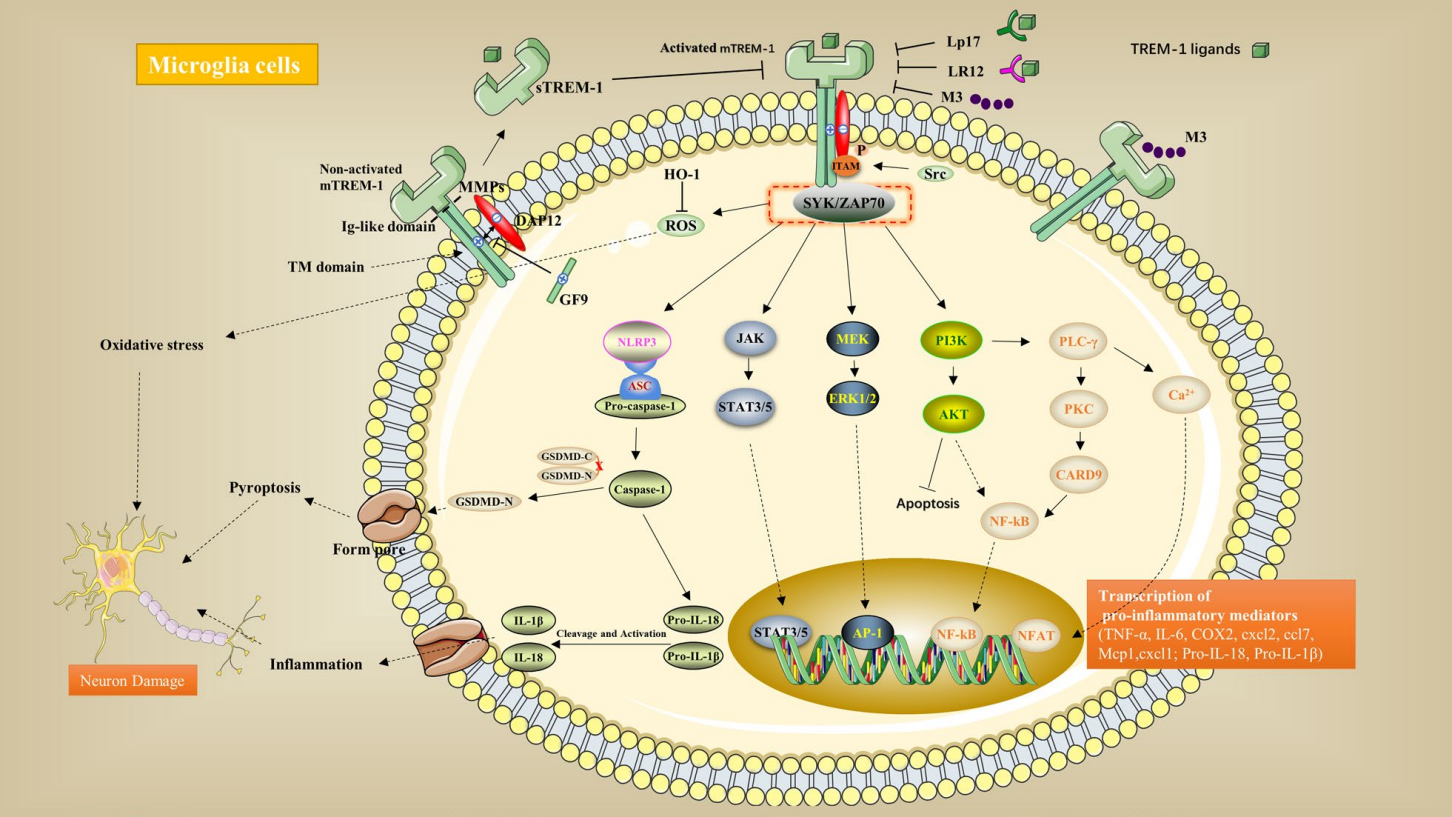
TREM-1 signaling pathway. Two isoforms of TREM-1 exist: the membrane-bound type (mTREM-1) and the soluble type (sTREM-1). mTREM-1 is located on the plasma membrane, and its extracellular immunoglobulin(Ig)-like domain is responsible for recognizing ligands such as pathogen-associated molecular patterns and danger-associated molecular patterns. After activation, the transmembrane (TM) domain transmits signals intracellularly by binding to the adapter molecule DAP12. Upon binding, tyrosine phosphorylation in immunoreceptor tyrosine-based activation motif (ITAM) that is mediated by Src-family kinases recruits the zeta-associated protein of 70 kDa (ZAP70) and spleen tyrosine kinase (SYK), thereby initiating a cascade of downstream signalization, including the activation of the phosphoinositide phospholipase C-gamma (PLCγ), phosphoinositide 3-kinases (PI3K), Janus kinase (JAK), and mitogen-activated protein kinases (MAPK) pathways. These pathways induce Ca^2+^ mobilization, actin cytoskeleton rearrangement, and activation of transcription factors, which are responsible for encoding the expression of inflammatory mediators. Metalloproteinases (MMPs) can cleave mTREM-1 into the soluble form, which competes with mTREM-1 for the same ligands and blocks mTREM-1 signaling. Decoy peptides that have been developed for the TREM-1 ligand-binding domain include LP17, LR12, and M3, while GF9 is an inhibitor that targets the transmembrane structural domains of TREM-1. All of these can block mTREM-1 and inhibit the activity of the inflammatory pathway. In addition to the inflammatory pathway, TREM-1 induces pyroptosis through the NLRP3/Caspase-1 signaling pathway and participates in oxidative stress through ROS release. The above pathways together contribute to neuronal damage

Acute ischemic stroke is a major cause of disability and death in adults. It is mainly caused by atherosclerosis and thromboembolism. Microglia are activated after a stroke and can induce the secretion of proinflammatory cytokines that result in neuroinflammation and cause the progression of ischemic brain injury [82]. The detection of biomarkers involved in the neurological injury process may help to assess prognosis. In a prospective cohort study that included 50 patients with acute ischemic stroke, Backes et al. [83]. evaluated the feasibility of using serum TREM-1 and TREM-2 as potential biomarkers for acute ischemic stroke. Serum levels of TREM-1 and TREM-2 were found to be elevated in patients after stroke. There was a correlation between TREM-1 with the modified Rankin Scale (mRS) and National Institutes of Health Stroke Scale (NIHSS) within 24 h. Patients with a poor outcome (mRS > 2) at hospital discharge had significantly higher serum TREM-1 concentrations within 24 h of onset. This study suggests that elevated TREM-1 within 24 h of onset correlates with stroke severity and is an independent prognostic factor for stroke. However, TREM-1 and TREM-2 levels did not correlate with neurological prognosis after 4 years. The sample size in this study was relatively small, and a larger sample size is needed to further validate the results. Liang et al. [84]. found upregulation of TREM-1 on microglia after cerebral infarction in a rat MCAO model. LP17 reduced infarct volume, decreased brain oedema, inhibited microglial activation, and reduced neuronal damage. Both in vivo and in vitro experiments have shown that inhibition of TREM-1 decreases reactive oxygen species (ROS) and malondialdehyde levels, enhances superoxide dismutase (SOD) activity, and increases haem oxygenase-1 (HO-1) levels. In LP17-treated animals, the mRNA levels of TNF-α, cyclooxygenase-2 (COX-2) and IL-6 are decreased, and IL-10 expression is increased. In a pyroptosis-related study, NOD-, leucine-rich repeat (LRR)- and pyrin domain-containing protein 3 (NLRP3), caspase-1, gasdermin D (GSDMD), ASC (an apoptosis-associated speck-like protein containing a CARD), IL-1β and IL-18 levels were found to be elevated after cerebral infarction, while LP17 decreased their expression. This suggests that TREM-1 can exacerbate brain damage by promoting microglial activation, participating in oxidative stress via ROS, and inducing pyroptosis via NLRP3/caspase-1 (Fig. 1). TREM-1 has also been found to be expressed on microglia in a mouse MCAO model. Inhibition of TREM-1 inhibits CARD9/NF-κB-mediated inflammation and NLRP3/caspase-1-mediated microglial pyroptosis, as well as neutrophil infiltration. Caspase-1 activated by SYK can cleave GSDMD to release n-terminal fragments, which forms pores on microglia and leads to the extracellular release of IL-1 and IL-18. Microglial pyroptosis and neutrophil infiltration may be important factors in exacerbating neuroinflammation. Inhibition of TREM-1 not only ameliorates ischemia-induced neural damage but also promotes cell proliferation in the hippocampal region, enhances synaptic plasticity, and improves long-term neurobehavioural function in mice, providing a new direction for the treatment of ischemic stroke [85]. Liu et al. [86]. found that when peripheral blood CD11b( +) CD45( +) myeloid cells were transferred to the ischemic brain, TREM-1 expression was induced within a few hours after stroke. Positron emission tomography and computerized tomography imaging of mice using radioactive ^64^Cu-labelled TREM-1 antibody as a tracer showed that the TREM-1 signal was significantly more pronounced in the cortex and striatum on the infarcted side compared to the contralateral side in addition to its significantly higher expression in the spleen and intestine. Previous studies have shown that sympathetic activation after stroke increases intestinal permeability [87, 88], promotes bacterial translocation, and causes bacteremia. In one study, TREM-1 was found to be produced in intestinal and peripheral monocytes and macrophages induced by intestinal barrier disruption and bacterial translocation after MCAO. TREM-1 expression in the peripheral blood and spleen also decreased after intestinal permeability was decreased with adrenergic inhibitors. Additionally, TREM-1 activation in the intestine exacerbates intestinal epithelial permeability, thereby further contributing to bacterial translocation across the intestinal barrier, which is associated with the development of bacteremia after stroke. Researchers have used genetic and pharmacological approaches to inhibit TREM-1 expression and found reduced brain infarct size and increased survival in mice. It is hypothesized that targeting TREM-1 treatment may reduce ischemic brain injury and stroke-associated infections. Poststroke depression (PSD) is a common poststroke complication that exacerbates cognitive impairment, impairs neurological recovery, and reduces quality of life. Some investigators have found that sTREM-1 and glial cell-derived neurotrophic factor levels are reduced in the peripheral blood of PSD patients compared to nondepressed poststroke patients, and are negatively correlated with the severity of depressive symptoms, suggesting that glial cells (i.e., astrocytes and microglia) may be involved in the development of depressive symptoms in the early poststroke period [89].

### Subarachnoid hemorrhage

SAH is mainly caused by aneurysm rupture. Its morbidity and mortality rates are high, and the inflammatory response is the main factor that causes poor prognosis in patients [90, 91]. Sun et al. [92–94]. showed significantly elevated levels of sTREM-1 in cerebrospinal fluid (CSF) in patients with SAH, the extent of which correlated with the intensity of the inflammatory response and the severity of disease, suggesting that TREM-1 may be involved in initiating and amplifying the inflammatory cascade process in early brain injury (EBI) after SAH. They hypothesized that sTREM-1 could be used as a biologic marker to assess the severity and prognosis of SAH. Additionally, plasma sTREM-1 concentrations are elevated in patients with aneurysmal SAH and are positively correlated with SAH severity and C-reactive protein (CRP) levels [95]. After SAH, in addition to the activation of resident immune cells in the brain, neutrophils also rapidly infiltrate the brain parenchyma and contribute to the mediation of neuroinflammation [96, 97]. In an experimental SAH model, TREM-1 interacted with SYK to activate the CARD9/NF-κB pathway and the PAD4/NETs pathway, the former triggering the proinflammatory subtypes of microglia and inducing neuroinflammation. Neutrophil extracellular traps (NETs) can also cause inflammation and promote M1 microglial polarization, exacerbating the inflammatory response [97, 98]. Blockade of TREM-1 attenuates microglial inflammation-related injury and inhibits the mutual interference between peripheral and central immunity [95]. Sun et al. [94]. demonstrated in an EBI model induced by SAH that intracerebral TREM-1 started to increase at 6 h and peaked at 48 h in microglia and vascular endothelial cells. The expression of TLR4, MyD88, and NF-κB decreased after TREM-1 inhibition by LP17, while the opposite result was observed in the TREM-1 agonist group. Inhibition of TLR4 decreased TREM-1 levels and significantly improved neurological scores, brain oedema, and blood‒brain barrier disruption. This study shows that crosstalk exists between TREM-1 and TLR4 pathways, which jointly promote inflammation and exacerbate EBI. In another study, Sun et al. [99]. demonstrated that the protective effect of LP17 on EBI was dose-dependent and was associated with the inhibition of downstream p38MAPK/MMP-9 expression and reduced degradation of zonula occludens-1 (ZO-1). Xu et al. [100] found that TREM-1 induced microglial pyroptosis and aggravated neuroinflammation via NLRP3. Inhibition of TREM-1 reduced microglial activation and pyroptosis, inhibited neutrophil infiltration and neuronal apoptosis, and reduced brain oedema and blood‒brain barrier disruption, thereby improving brain function. Therefore, targeting TREM-1 may be a potential approach to the treatment of SAH.

### Intracerebral hemorrhage

Spontaneous intracerebral hemorrhage (ICH) is a common neurological disease caused by the spontaneous rupture of intracranial vessels. It is mostly associated with hypertension and cerebrovascular malformations and has the highest mortality rate among cerebrovascular diseases. The release of inflammatory mediators induced by microglial activation is involved in secondary brain injury after ICH [101]. After brain injury, microglia initially polarize into the M1 phenotype and secrete inflammatory factors that are involved in the brain injury process. Within 7 days after ICH, microglia polarize into the M2 phenotype, which contributes to haematoma clearance and brain injury repair [102]. Therefore, modulation of the microglial phenotype may be a new target for brain hemorrhage therapy. TREM-1 expression is elevated in an experimental rat brain hemorrhage model [103]. In a mouse model of ICH, the expression of HMGB1, TREM-1, PKC and CARD9 was elevated. TREM-1 is expressed on microglia. Inhibition of TREM-1 ameliorates neurobehavioural deficits, reduces brain oedema, decreases M1 phenotype microglia around the haematoma, promotes microglial polarization into the M2 phenotype, and inhibits proinflammatory cytokine release. CARD9 activation reverses the effects of LP17. Administration of TREM-1 monoclonal antibody increases neurobehavioural deficits and proinflammatory cytokine release and decreases the number of M2 microglia, and inhibition of PKCδ reverses these results. It is hypothesized that TREM-1 may regulate microglial polarization and affect neuroinflammation through the PKCδ/CARD9 signaling pathway. In addition, the TREM-1 interaction with HMGB1 increases after ICH. Inhibition of HMBG1 decreases neuroinflammation, and this effect is reversed by activating TREM-1. Researchers have proposed that HMGB1 may be a ligand for TREM-1 [59]. Clinical investigations have found elevated serum sTREM-1 concentrations in patients with primary cerebral hemorrhage, which may be used to assess the severity of disease and the prognosis of patients with ICH [104]. In a transcriptome analysis of peripheral whole blood from 18 patients with ICH, 440 genes associated with ICH were identified, and were mainly expressed in inflammatory pathways, including NF-κB, TREM-1, and other neuroinflammatory signaling pathways. Therefore, the investigators proposed that the pathways and genes identified in their study may be therapeutic targets for cerebral hemorrhage [105].

### Parkinson’s disease

PD is one of the most common neurodegenerative diseases. Its prevalence increases with age, with a prevalence of 1% in people aged 60 years or older [106]. In China, the prevalence in people over 60 years of age was 1.37% in 2015, with a total of approximately 3.62 million patients [107]. Neuroinflammation mediated by microglial hyperactivation has been reported to be involved in the pathological process of neurodegenerative diseases [108]. Microglia are overactivated in the brain tissue of PD patients, and a large number of proinflammatory molecules can be detected in the brain tissue and CSF of patients [109]. However, the molecular mechanisms of microglia in the progression of PD have not been studied extensively. Feng et al. [110] investigated the role of TREM-1 in PD models and found that the LPS-induced upregulation of nitric oxide synthase (iNOS), cyclooxygenase-2 (COX-2) and NF-κB protein in BV2 cells and the downregulation of IkappaB (IκB) protein levels were reversed by LP17. In addition, LP17 enhanced the LPS-induced expression of autophagy-related proteins, and transfection of small interfering RNA (siRNA) in BV2 cells to inhibit TREM-1-attenuated inflammation and enhanced autophagy. LP17 also alleviates zebrafish dyskinesia and downregulates TREM-1, iNOS, and calpain-1 mitochondrial RNA (mRNA) expression in 6-hydroxydopamine (6-OHDA)-induced zebrafish PD models while also reversing the downregulation of tyrosine hydroxylase (TH) protein expression and the upregulation of TNF-α protein expression induced by 6-OHDA. LP17 significantly attenuates 6-OHDA-induced glial cell hyperactivation in rat PD models. Therefore, inhibition of TREM-1 could play a neuroprotective role by enhancing autophagy and attenuating neuroinflammatory responses.

### Alzheimer’s disease

AD is the most common neurodegenerative disease and the most common cause of dementia. Aβ deposition in the brain parenchyma and neurogenic fibre tangles are the main causes of AD. Jiang et al. [54]. found increased amyloid deposition in the mouse brain after TREM-1 inhibition by intracerebral injection of lentiviral particles, whereas TREM-1 overexpression by a lentiviral strategy or agonistic antibodies alleviated AD neuropathology and improved AD-related spatial cognitive deficits. This suggests that TREM-l regulates the progression of AD by promoting microglial phagocytosis of Aβ. Sao et al. [111]. found significantly elevated TREM-1 mRNA levels in the blood of AD patients. Additionally, plasma sTREM-1 concentrations are significantly elevated in AD patients and positively correlated with disease progression and total tau protein levels in plasma [112]. In patients with mild cognitive impairment and AD, the TREM-1 mutant rs2234246 is closely associated with brain amyloid deposition [113]. It is hypothesized that TREM-1 may be involved in AD by influencing neuropathology, but the exact mechanism is not clear. Neuroinflammation plays an important role in the development of AD [114]. Patients with AD have elevated levels of IL-1, IL-6, TNF-α and interferon gamma (IFN-γ) in the brain, and activated microglia can be seen around amyloid plaques [115]. We hypothesize that TREM-1 may be involved in the progression of AD by inducing neuroinflammation. Tau oligomers (TauO) are considered the most neurotoxic tau proteins causing cognitive impairment [116]. Exposure of primary astrocytes to TauO induces HMGB1 release, which mediates astrocyte senescence and paracrine senescence in adjacent cells. Ethyl pyruvate (EP) and glycyrrhetinic acid (GA) inhibit HMGB1 release and prevent TauO-induced cellular senescence by inhibiting the activation of the p38MAPK and NF-κB signaling pathways and reducing TauO-associated senescence-associated secretory phenotype (SASP) levels and neuroinflammation. In addition, hTau transgenic mice develop tauopathy at 12 months of age, and EP + GA treatment significantly reduces TauO and the number of senescence-like cells and reduces neuroinflammation, thus improving cognitive function. TauO-induced HMGB1 release plays a key role in cellular senescence, chronic neuroinflammation and the progression of senescence-associated tau protein pathology. This may be an important pathological mechanism in AD [60]. The SASP includes the active secretion of various chemokines, inflammatory cytokines and proteases and is an indicator of human ageing [117]. HMGB1 is a recognized component of SASP [118]. TauO colocalizes with astrocytes and microglia in the brains of AD patients, and TauO can trigger chronic neuroinflammation [119]. Translocation of HMGB1 from the nucleus to the cytoplasm in TauO-associated astrocytes and microglia is a feature of cellular senescence [120, 121]. Whether TauO has similar effects on microglia as it does on astrocytes, and whether tauO-induced chronic neuroinflammation is associated with the TREM-1 pathway activated by HMGB1 are questions that require further study.

### Gliomas

Gliomas are the most common primary brain tumours, with glioblastomas being the most malignant. It has been reported that in patients with high-grade gliomas (GIII and GIV), patients with a TREM-1/TREM-2 ratio above 125 survived a significantly shorter amount of time than those with a ratio less than 125 [122]. This finding could aid in the prediction of prognoses for glioma patients. In addition, contrary to most findings that sTREM-1 concentrations are elevated in tumour tissues, serum sTREM-1 levels are lower in glioma patients than in healthy volunteers [122], though this result needs to be confirmed by expanding the sample size.

### CNS infections

The common causative agents of bacterial meningitis are Streptococcus pneumoniae, Neisseria meningitidis, and Haemophilus influenzae. The mortality rate of this disease is high, and an early, rapid, and accurate diagnosis of acute bacterial meningitis is important to improve patients’ prognoses. However, many patients lack typical clinical manifestations, and CSF culture may show false negative results, which makes early diagnosis difficult. Some studies have found that sTREM-1 levels in CSF may contribute to the diagnosis of bacterial meningitis. Liu et al. [123]. found that the combined detection of sTREM-1 and decoy receptor 3 (DcR3) was more accurate than the detection of sTREM-1 or DcR3 alone. Determann et al. [124]. performed a retrospective cohort analysis and showed elevated levels of sTREM-1 in the CSF of patients with bacterial meningitis and demonstrated that its concentration correlates with mortality and could help predict prognosis. In another prospective study, sTREM-1 was similarly found to be elevated in the CSF of patients with bacterial meningitis with high specificity. However, contrary to the findings of Determann et al. sTREM-1 concentrations have also been shown to be higher in surviving patients than in deceased patients [125]. Due to the small sample size, a large prospective study is needed to confirm the role of sTREM-1 in the diagnosis and prognosis of bacterial meningitis. Additionally, elevated sTREM-1 may be used to aid in the diagnosis of external ventricular drain-related ventriculitis and to distinguish between colonization and infection [126].

### Other CNS diseases

SCI is a serious neurological disease. It includes both primary and secondary injuries. SCI has a high disability rate and poor neurological recovery, which significantly affects the quality of survival of patients and increases the socioeconomic burden. In a mouse model of SCI, TREM-1 protein and mRNA levels were elevated, the number of spinal cord neurons was reduced, microglia and astrocytes were activated, and TREM-1 knockout mice exhibited better motor function and reduced expression of inflammation-related factors. In addition, TREM-1 inhibition decreased oxidative stress markers and increased antioxidants such as superoxide dismutase-1 (SOD1), nicotinamide adenine dinucleotide plus hydrogen (NADH) quinone oxidoreductase 1 (NQO-1), HO-1, and Nrf2. The same results were obtained in LPS-stimulated astrocytes and BV2 cells by transfection with TREM-1 siRNA, and the aforementioned reduction in inflammation, oxidative stress, and microglial activation after TREM-1 inhibition was abolished by the use of the HO-1 inhibitor. This demonstrates that inhibition of TREM-1 attenuates inflammation and oxidative stress, thereby improving SCI [127]. SCIRI can induce free radical release, inflammatory factor production, and enhanced endoplasmic reticulum stress, leading to irreversible neurological damage. In a mouse model of SCIRI, spinal cord neuronal apoptosis is exacerbated, and TREM-1 protein and mRNA expression are significantly upregulated. SYK expression is downregulated and neuronal apoptosis is reduced after intrathecal injection of short hairpin RNA (shRNA) adenovirus to knock down TREM-1 expression. An oxygen-glucose-serum deprivation/recovery (OGSD/R) model was established with N2a cells to simulate the pathophysiological process of SCIRI in vitro. Under OGSD/R conditions, TREM-1 expression was significantly elevated, SYK phosphorylation levels were increased, the proapoptotic factors caspase-3 and Bax were upregulated, and the apoptosis inhibitor B-cell lymphoma-2 (Bcl2) was downregulated. Furthermore, after transfection with siRNA, caspase-3, Bax, and Bcl2 protein levels were restored, and the TREM-1 interaction with SYK was found to mediate neuronal apoptosis through the activation of the PI3K/AKT and NF-κB signaling pathways by immunoprecipitation and immunofluorescence [128]. Under chronic social stress conditions, microglia are activated in mouse hippocampal neurons, thereby increasing the number of typical pathways associated with inflammation, including TREM-1 signaling [129]. It has been hypothesized that TREM-1 may be associated with chronic social stress disorder, though the exact mechanism needs to be studied in depth.

## Conclusion and prospective research avenues

In summary, the TREM-1 signaling pathway in CNS diseases exacerbates brain damage by participating in neuroinflammation. Blocking TREM-1 can inhibit the release of inflammatory mediators and improve prognosis. To date, peptide blockers developed for the TREM-1 ligand-binding structural domain include LP17, LR12, and M3, of which nangibotide (also known as LR12) has been evaluated for safety and pharmacokinetics in a phase IIa clinical trial in patients with septic shock [130]. A phase IIb randomized controlled trial is currently underway in patients with septic shock to further validate efficacy, safety and tolerability [131]. GF9 is a TREM-1 inhibitor that targets transmembrane structural domains that was discovered by Sigalov et al. [132], and its effectiveness has been demonstrated in animal models of arthritis [133], alcoholic liver disease [134], pertussis [135], and pancreatic cancer [136]. The efficacy achieved by TREM-1 inhibitors in animal experiments lays the foundation for the development of immunomodulatory strategies in human patients, and TREM-1 may become a new target for the treatment of CNS diseases.

However, there are still many limitations in previous studies. For example, the exact mechanism of TREM-1 in the inflammatory cascade is not yet fully understood. In addition, current studies of TREM-1 in human CNS diseases are mainly observational studies with small sample sizes and limited mechanistic studies. Furthermore, there are few studies on the source and nature of TREM-1 ligands and their expression in CNS disease processes. Finally, the interplay between central and peripheral immunity mediated by TREM-1 in CNS diseases has not been thoroughly investigated. Elucidation of the origin and identity of ligands, the molecular mechanisms of receptor‒ligand interactions and the regulation of signal transduction will contribute greatly to our understanding of TREM-1 biology and provide a basis for the development of novel and more effective targeted therapeutic agents based on endogenous ligands. We expect that a large number of TREM-1 modulators with clinical application potential will emerge in the near future. In addition, the diagnostic and prognostic value of sTREM-1 in CNS diseases needs to be supported by studies with larger samples to provide more evidence.

## Data Availability

Not applicable.

## Abbreviations

TREM-1: Triggering receptor expressed on myeloid cells-1
TREMs: Triggering receptors expressed on myeloid cells
TREM-2: Triggering receptor expressed on myeloid cells-2
DAP10: DNAX-activating protein 10
DAP12: DNAX-activating protein 12
MMPs: Metalloproteinases
CNS: Central nervous system
SAH: Subarachnoid hemorrhage
SCI: Spinal cord injury
PD: Parkinson’s disease
AD: Alzheimer’s disease
ITAM: Immunoreceptor tyrosine-based activation motif
ZAP70: Zeta-associated protein of 70 kDa
SYK: Spleen tyrosine kinase
PLCγ: Phosphoinositide phospholipase C-gamma
PI3K: Phosphoinositide 3-kinases
Akt: Protein kinase B
JAK: Janus kinase
MAPK: Mitogen-activated protein kinases
HIFs: Hypoxia-inducible transcription factors
Ig-like domain: Immunoglobulin-like domain
TM domain: Transmembrane domain
mDCs: Mature dendritic cells
iDCs: Immature dendritic cells
mTOR: Mammalian target of the rapamycin
IL-8: Interleukin 8
TNF-α: Tumor necrosis factor alpha
PGE2: Prostaglandin E2
PGN: Peptidoglycan
LPS: Lipopolysaccharide
WARS1: Tryptophanyl-tRNA synthetase 1
CpG-ODN: CpG oligodeoxynucleotides
PGD2: Prostaglandin D2
PGJ2: Prostaglandin J2
TLR: Toll-like receptor
15-dPGJ2: 15-Deoxy-delta-prostaglandin J2
Nrf2: Nuclear factor erythroid 2-related factor 2
NF-κB: Nuclear factor kappa B
IL-10: Interleukin 10
TGF-β: Transforming growth factor β
HMGB1: High-mobility group box 1
HSP70: Heat shock protein 70
PGLYRP1: PGN recognition protein 1
PRRs: Pattern recognition receptors
SPR: Surface plasmon resonance
eCIRP: Extracellular cold-inducible RNA-binding protein
rmCIRP: Recombinant murine CIRP
I/R: Ischemia/reperfusion
NOD: Oligomerization domain
NLRs: NOD-like receptors
KCs: Kupffer cells
mRS: Modified Rankin Scale
NIHSS: National Institutes of Health Stroke Scale
MCAO: Middle cerebral artery occlusion
ROS: Reactive oxygen species
PSD: Post-stroke depression
CSF: Cerebrospinal fluid
CRP: C-reactive protein
EBI: Early brain injury
ZO-1: Zonula occludens-1
NLRP3: NOD-, leucine-rich repeat (LRR)- and pyrin domain-containing protein 3
PKCδ: Protein kinase C-delta
CARD9: Caspase recruitment domain family member 9
COX-2: Cyclooxygenase-2
iNOS: Nitric oxide synthase
IκB: I kappa B
siRNA: Small interfering RNA
mRNA: Mitochondrial RNA
6-OHDA: 6-Hydroxydopamine
TH: Tyrosine hydroxylase
Aβ: Amyloid beta
IFN-γ: Interferon gamma
TauO: Tau oligomers
SASP: Senescence-associated secretory phenotype
DcR3: Decoy receptor 3
SOD: Superoxide dismutase
NADH: Nicotinamide adenine dinucleotide plus hydrogen
NQO-1: Quinone oxidoreductase 1
HO-1: Heme oxygenase-1
SCIRI: Spinal cord ischemia–reperfusion injury
shRNA: Short hairpin RNA
OGSD/R: Oxygen-glucose-serum deprivation/recovery
Bcl2B: Cell lymphoma-2
IRAK3: Interleukin-1 receptor activated kinase
GSDMD: Gasdermin D
PAMP: Pathogen-associated molecular patterns
DAMP: Danger-associated molecular patterns
ERK: Extracellular signal-related kinase
RAGE: Receptor for advanced glycation end-products
EP: Ethyl pyruvate
GA: Glycyrrhetinic acid
